# Advancing Tissue Engineering Through a Portable Perfusion and Incubation System

**DOI:** 10.3390/bioengineering12050554

**Published:** 2025-05-21

**Authors:** Angie Zhu, Emmett Reid, Tilak Jain, Amatullah Mir, Usmaan Siddiqi, Olivia Dunne, Narutoshi Hibino

**Affiliations:** 1 Section of Cardiac Surgery, Department of Surgery, University of Chicago, 5841 S. Maryland Ave., Chicago, IL 60637, USA; ereid@uchicago.edu (E.R.); amir1@uchicago.edu (A.M.); usmaansiddiqi@yale.edu (U.S.); olivia.dunne@einsteinmed.edu (O.D.); narutoshi.hibino@bsd.uchicago.edu (N.H.); 2 37degrees, 111 North Wabash Ave. Ste. 100, Chicago, IL 60602, USA; tilak.jain@37-degrees.com; 3 Pediatric Cardiac Surgery, Advocate Children’s Hospital, 4440 W 95th St., Oak Lawn, IL 60453, USA

**Keywords:** perfusion, tissue engineering, biotransport systems, bioreactor, heart in a box

## Abstract

Perfusion offers unique benefits to tissue-engineered systems, enhancing oxygen and nutrient transport, which improves tissue formation and growth. In this study, we present a novel and integrated portable perfusion system. Weighing < 10 lbs, the system can maintain continuous flow in a standard incubation environment (37 °C, 5% CO_2_), effectively functioning as a portable perfusion and tissue culturing system. To characterize the perfusion system’s flow parameters, we measured the volumetric flow rate across a range of pressures and found that the system could achieve flow velocities between 1.69 to 4.6 μm/s, which is similar to in vivo interstitial flow. Computational fluid dynamics revealed fully developed laminar flow within the sample-containing region of the perfusion system, helping ensure even fluid and nutrient distribution. To study the system’s compatibility with live tissues, bioengineered tissue patches were created and perfused. After 24 h of perfusion, no significant difference in cell viability was observed between the perfused samples and static controls, indicating no adverse effects on cell health. Perfusion also facilitated enhanced spatial organization within tissue patches, reducing the inter-spheroids distance. Furthermore, perfusion strengthened the tissue matrix and reduced the degradation rate of the hydrogel scaffold. Complemented by its ability to provide mobile perfusion and incubation, this novel integrated portable perfusion system holds promise for promoting tissue maturation and advancing tissue bioengineering studies.

## 1. Introduction

### 1.1. Background

Tissue-engineered systems, composed of three-dimensional (3D) stem cell composites reinforced with an extracellular matrix, present promising avenues for tissue regeneration [1]. However, key concerns to clinical implementation include (1) sustained cell viability, (2) hypoxia and accumulation of toxic metabolites [2], and (3) development of appropriate tissue vasculature to recapitulate in vivo structures [3].

Perfusion offers enhancements to these challenges; perfused conditions have been found to improve the spatial uniformity of cell distribution, enhancing the expression of specific cell markers within the core of 3D tissue aggregates [4]. In comparison, static studies have demonstrated cell-specific markers predominantly on the periphery of tissues, reflecting the presence of nutrient concentration gradients associated with diffusional mass transport [5]. As such, perfusion facilitates increased delivery of oxygen and nutrients to the center of tissues, combating the hypoxia concerns intrinsic to the 3D nature of engineered tissues. Additionally, the fluidic movement of perfusion systems generates mechanical stimulation, creating a dynamic environment that enhances the generation of vasculature and tissue development [6].

Perfusion systems can generally be classified as microfluidic, interstitial, or organ, with each targeting a specific construct. Microfluidic perfusion systems target single organoids with sub-microliter volume flow rates [7]. Interstitial systems aim to mimic biological interstitial flow rates in tissues. Finally, large organ perfusion generally tailors to larger models, such as whole organs [8]. However, similar limitations arise across these systems. Because perfusion often requires the usage of external pump systems or incubation systems, perfusion generally lacks mobility which makes transportation of engineered tissue difficult and increases the size burden. Moreover, individual laboratories must often design their own perfusion systems using available tools. While this allows researchers to tailor their systems to their unique needs, this also limits the reproducibility of studies across different facilities.

### 1.2. Design of the FluidON and the Integrated System Components

**Figure 1:**
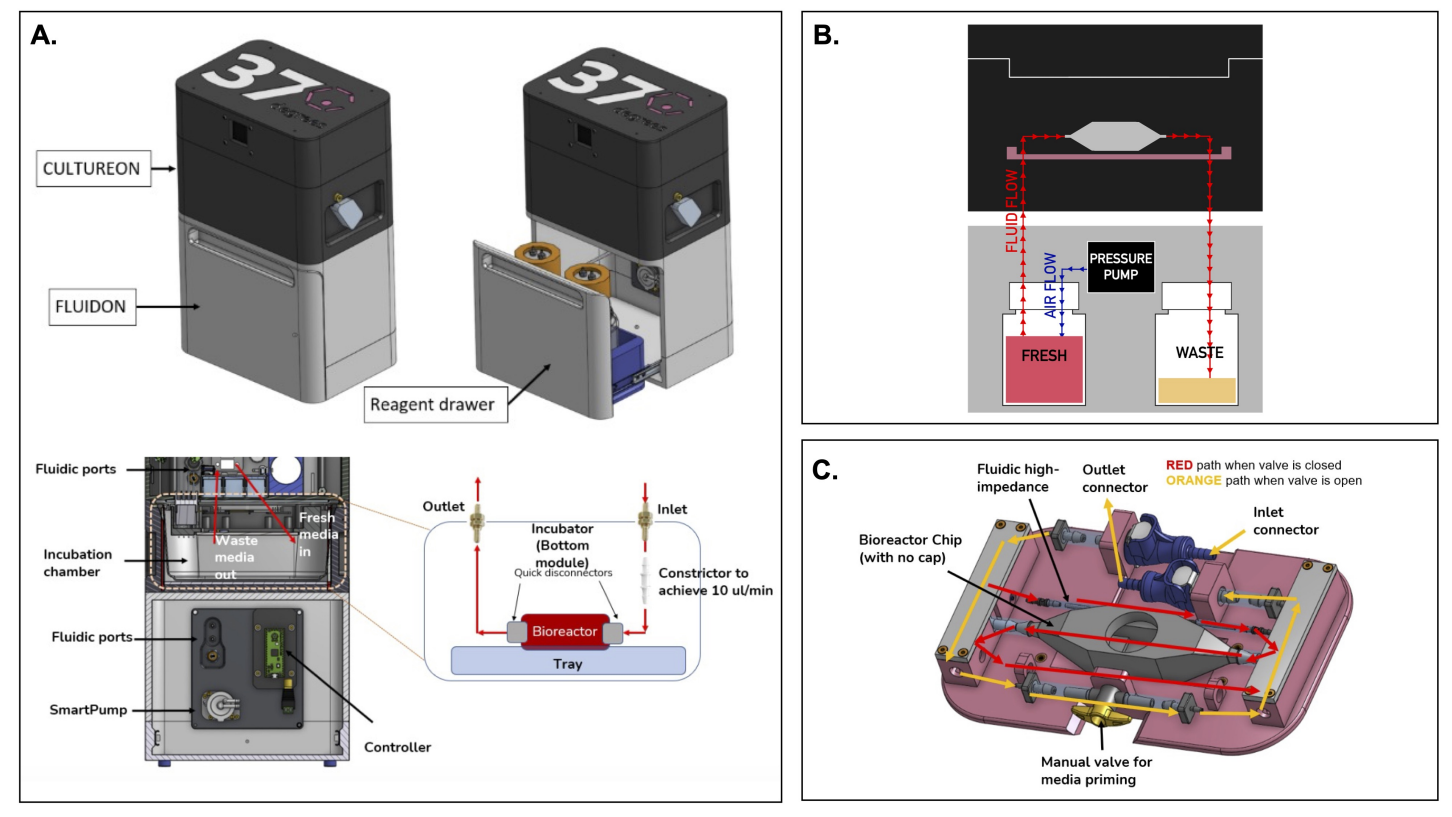
An overview of the integrated portable perfusion system. (**A**) As shown by a computer-aided design (CAD) of the perfusion system, the CultureON and the FluidON are integrated. A reagent draw in the FluidON hosts the fresh and waste bottles while a Smartpump allows for continuous media flow. (**B**) A simplified perfusion system schematic demonstrates how pressure generated in the FluidON routes media flow into the CultureON, where the bioreactor houses the tissue. (**C**) Represented in a CAD, a custom tray and bioreactor were designed. Depending on the valve orientation, two flow paths are possible.

A portable perfusion system is still missing. In this study, we present a novel, portable, and modular perfusion system that utilizes precise pressure control to achieve consistent media flow (Figure 1A). Generally, pressure-based systems connect a perfusion chamber in series with two reservoirs. The fresh reservoir begins filled with media while the waste reservoir begins empty. Perfusion occurs as air enters the fresh reservoir and applies pressure on the surface of the media in the bottle. This process drives the media to flow out of the reservoir, through tubing channels, into the perfusion bioreactor, and ultimately into the waste reservoir.

The perfusion system we devised—called the FluidON—is similarly pressure-based (Figure 1B). To create the FluidON, we utilized a piezoelectric air pump that drives filtered air (1.2 microns) into a fresh reagent bottle. A 100-micron bore (I.D) 4-inch tubing constrictor tunes the media flow, providing high-fluidic impedance and enabling low flow rates. Media continuously circulates through the FluidON system and is then collected through an outlet into the waste container.

Furthermore, media flow is routed to the CultureON (37degrees, Inc.; Chicago, IL, USA)—a portable CO_2_ incubator module. The CultureON contains a bioreactor and a bioreactor tray assembly, both of which are described in detail subsequently. The CultureON module is able to control the temperature at 37 °C and 5% CO_2_, maintaining the bioreactor components at standard incubation conditions. Humidity, temperature, and CO_2_ levels can be monitored and regulated through external software. The CultureON module is designed to stack vertically on the FluidON module, which contains a reagent drawer that houses the fresh media and waste bottles. The total dimensions of the system fit within 14×9×6 inch, and both modules weigh <5 lbs individually. To allow for the portability of the integrated FluidON and CultureON systems, we utilized disposable CO_2_ gas cartridges to remove the need for external gas supply lines when culturing. Additionally, the integrated system can be direct current (DC) battery-powered, allowing the system to be dismounted from wall outlets and transported while maintaining full functionality.

Tissue samples are placed within a bioreactor inside the integrated FluidON and CultureON system. The bioreactor is composed of two components: the main chamber and the lid. Before perfusion, the tissue sample is loaded into the chamber, and the lid is secured with parafilm to create an airtight seal that holds the two components together. The main chamber can hold a 10×10×10 mm (1 cm^3^) tissue in the center compartment. The inner compartment is fitted with posts that hold the sample in place. The two walls of the inner compartment that face the inlet and outlet ports are porous, so fluid can easily perfuse through inserted tissues without shifting their position.

A custom-designed tray (37degrees, Inc.; Chicago, IL, USA) houses the bioreactor (Figure 1C). Once the inlet and outlet connections are made, the manual valve can be opened or closed. When the valve is open, media flow bypasses the high-impedance fluidic resistor and the bioreactor for fast fluid line priming of the entire system. This fills the fluid lines and ensures bubbles are removed from the system. When the manual valve is closed, the media flows through the high-fluidic impedance, which significantly reduces the flow rate and passes media through the bioreactor. This setup can maintain fluid flow and incubation for several days, with the initial fresh media volume acting as the limiting reagent.

The current model of the FluidON contains media bottles that can be disconnected for sterilization through a high-temperature autoclave. We perfused 70% ethanol throughout the system for 15 min to sterilize other remaining non-autoclavable components. We observed no contamination after 72 h of perfusion under standard incubation conditions.

## 2. Materials and Methods

Materials for cell culture––Dulbecco’s Modified Eagle Medium (DMEM), fetal bovine serum (FBS), and penicillin/streptomycin (P/S)—were procured from Thermo Fisher Scientific (Waltham, MA, USA), and Phosphate-Buffered Saline (PBS) was obtained from Sigma-Aldrich (Saint Louis, MO, USA). For spheroid creation, pipetting reservoirs were purchased from Cole-Parmer (Vernon Hills, IL, USA) and 96-well (U) bottom plates were obtained from IWAKI (Castle Hill, Australia). The dissociation reagents Type 2 collagenase from Worthington Biochemical Corporation (Lakewood, NJ, USA) and trypLE from Thermo Fisher Scientific were used. Mouse endothelial cells (mEC; C166-GFP) were obtained from ATCC (Manassas, VA, USA). Mouse mesenchymal stem cells (mMSCs) were kindly provided by Dr. Tong-Chuan He (University of Chicago, Biological Sciences Division; Chicago, IL, USA).

Viability was measured with LIVE/DEAD™ Viability/Cytotoxicity Kit and LIVE/DEAD™ Fixable Blue Dead Cell Stain Kit from Thermo Fisher Scientific. Used for fixation and permeabilization, 10% neutral buffered formalin was obtained from Thermo Fisher Scientific, while TrixonX and bovine serum albumin (BSA) were acquired from Sigma-Aldrich. Flow cytometry was performed in 12 × 75 mm tubes from Thermo Fisher Scientific.

To create hydrogels, thrombin and fibrin were obtained from Sigma-Aldrich. 10 × 10 × 5 mm vinyl specimen mold from Sakura Finetek USA (Torrance, CA, USA) were used. Custom molds were made with polydimethylsiloxane (PDMS) from a Krayden Dow Sylgard 184 Silicone Elastometer Kit obtained from Thermo Fisher Scientific. Superfrost Plus Microscope Slides were purchased from Fisher Scientific (Hampton, NH, USA). Tissues were imaged on 35 mm glass bottom dishes obtained from Cellvis (Mountain View, CA, USA).

To process and analyze microscope images, Fiji (version 2.14.0/1.54f), was used. Images were formatted for clarity and visual consistency using Photoshop (version 25.12.0) from Adobe Inc. (San Jose, CA, USA). To visualize and analyze data, R and RStudio (version 2023.09.1+494) and Python (version 3.4.4) were used. For computational flow dynamic (CFD) simulations, ANSYS CFX (version 19.2) was utilized. To analyze flow cytometry results, FlowJo™ from BD Biosciences (Franklin Lakes, NJ, USA) was used.

### 2.1. Cell Culture

mMSCs and mECs were cultured in media constituted of DMEM, 10% FBS, and 1% P/S. Cultures were incubated at 37 °C and 5% CO_2_ for 3–4 days.

### 2.2. Spheroid Creation and Dissociation

Spheroids were made from mEC and mMSC cells. Each spheroid contained 33,000 cells at a ratio of 80% mEC to 20% mMSC. To create spheroids, a cell count was performed for both cell types, and the different cells were adjusted to their desired numbers. This, in addition to the desired volume of media (10% FBS and 1% P/S in DMEM), was added to a 100 mL pipetting reservoir. This solution was mixed until homogenous, and 200 μL of the mixture was added to each well of a 96-well (U) bottom plate.

The plate was incubated for 72 h at 37 °C and 5% CO_2_. Spheroids were checked visually under the Invitrogen EVOS microscope to certify that spherical, round structures of roughly 500 μm diameter had formed in all the wells. Spheroids were then embedded into hydrogels to create bioengineered tissues.

### 2.3. Creating Bioengineered Tissue Patches

First, a custom sample holder was made by placing a 14×14 mm glass slide and a 14×14 mm slice of PDMS with an 8 mm hole in the center into an oxygen plasma etcher. The slide and PDMS were then pressed together, enabling chemical adhesion. Due to its dimensional compatibility with our bioreactor and integrated glass bottom, this mold is well-suited for imaging applications.

To make bioengineered tissue patches, fibrinogen and thrombin from human plasma were diluted to 20 Units/mL and 40 mg/mL aliquots, respectively. Tissues were made by suspending roughly 100 spheroids in 75 μL of diluted thrombin before being combined with 75 μL of diluted fibrin. This mixture was pipetted into the glass and PDMS mold. Variability in the number of spheroids suspended may occur as spheroids adhere to the pipette tip, resulting in partial transfer.

While the previously described tissue patch creation methodology is optimal for microscopy, we also crafted larger bioengineered tissues for studies that may require more cells. To create bioengineered tissue patches for flow cytometry analysis, 300 spheroids were suspended in 500 μL of diluted thrombin before combination with 500 μL of diluted fibrin. This mixture was pipetted into a disposable 10×10×5 mm vinyl specimen mold. Once hydrogel was set, the vinyl mold was cut, and the tissue was carefully removed with an X-ACTO knife. For static conditions, the bioengineered tissue patch was incubated in a 35 mm glass bottom dish with 3 mL media. For perfusion, the bioengineered tissue was inserted into the bioreactor and subject to perfusion in the FluidON at 3.46 ± 0.04 μm/s.

### 2.4. Flow Cytometry and Live/Dead Assay

A collagenase digestion was used to obtain single-cell assays for flow cytometry. To dissociate spheroid-containing tissues, samples were transferred to a 6-well plate. 5 mL collagenase (255 U/mg dry weight, 2 mg/mL stock concentration) was added, and the plate was incubated for 24 h. Shear force was introduced post-incubation through pipetting up and down using a large orifice pipette tip. At this point, hydrogels were degraded, and spheroids were dissociated back into single cells.

Tissue patches post-dissociation were transferred to 5 mL 12×75 mm tubes and centrifuged to remove the supernatant. Cells were further washed and resuspended in 1 mL PBS. To stain for dead cells, 1 μL LIVE/DEAD™ Fixable Blue Dead Cell Stain Kit was added to each tube. Samples were then washed to remove excess stain and fixed with 1000 μL 10% neutral buffered formalin for 15 min. Fixed cells were then washed in 1 mL of PBS with 1% BSA. In an ice box, cells were brought to the Cytometry and Antibody Technology Core Facility (University of Chicago; Chicago, IL, USA) where the NovoCyte Penteon Flow Cytometer (Agilent; Santa Clara, CA, USA) was used. Populations were gated to include single cells and exclude debris. Dead cells exhibit stronger fluorescence in the LIVE/DEAD™ Fixable Blue Dead Cell Stain Kit, an amine-reactive dye. Therefore, a positive control was created by treating mMSC cells with 70% ethanol to induce cell death. This population was used to establish gating parameters for the test samples.

For Live/Dead analysis conducted through confocal microscopy, the LIVE/DEAD™ Viability/Cytotoxicity Kit was used following the manufacturer’s instructions.

### 2.5. Flow Characterization

We calculated the FluidOn’s volumetric flow rate (*V*) by obtaining the mass flow rate (*M*) of fluid perfused through the system over a range of drive pressures (1–3 psi). DMEM was perfused through the entire perfusion system, collected at the exit point, and weighed on a ZEO-50 milligram scale (American Weigh Scales; Cumming, GA, USA). Lower pressures required longer collection times to ensure a measurable volume.

*M* was converted to *V* by dividing by the density of DMEM (ρ, Equation (1)). The volumetric flow rate was calculated over a range of pressure gradients (ΔP) and plotted as a function of drive pressure ($P_{d}$, Equation (2)). Note that in reality, the hydrostatic pressure ($P_{hs}$) is a function of time due to the height of fluid over the inlet and outlet changing as fluid flows through the system. However, Equation (2) assumes that $P_{hs}$ in the fresh and waste media bottles make negligible contributions across ΔP (<2.2 ×$10^{-7}$ psi/mL of DMEM). The system’s high fluidic impedance decreases the hydrostatic pressure contributions even further.

(1) $$V=\frac{M}{\rho }$$

(2) $$\Delta P=\left(P_{atm}+P_{hs,fresh}+P_{d}\right)-\left(P_{atm}+P_{hs,waste}\right)=P_{d}+\left(P_{hs,fresh}-P_{hs,waste}\right)\approx P_{d}$$

The average flow velocities ($u_{avg}$) were calculated by dividing volumetric flow rates by the cross-sectional area ($A_{c}$) of the portion of the bioreactor where our samples were housed (Equation (3)). This method is only valid for systems with laminar flow, meaning that the Reynolds number (*Re*) must be less than 2000. We calculated the Reynolds numbers (Equation (4)) for each psi using $u_{avg}$, the density (ρ = 1009 ± 3 kg/m^3^) and dynamic viscosity (μ = 9.30 ± 0.34 × 10^−4^ Pa · s) of DMEM supplemented with FBS, and the bioreactor’s hydraulic diameter (D=0.435).

(3) $$u_{avg}=\frac{V}{A_{c}}$$

(4) $$Re=\frac{\rho u_{avg}D}{\mu }$$

Lastly, the flow velocity profile (*U*) was obtained by solving the Navier-Stokes equation for an incompressible Newtonian fluid under creeping flow. The pressure gradient across the sample-containing region was back-calculated from the maximum flow velocity at a driving pressure of 1 psi. The solution was calculated by summing the first 15 terms of the analytical solution to Equations (5) and (6).

(5) $$\mu \left(\frac{\partial^{2}u}{\partial y^{2}}+\frac{\partial^{2}u}{\partial z^{2}}\right)=\frac{dp}{dx}$$

(6) $$U_{x}\left(y,z\right)\;=\;\sum_{m=1,3,5,\ldots }^{\infty }\sum_{n=1,3,5,\ldots }^{\infty }\frac{16\;(-\frac{dp}{dx})}{\mu \;\pi^{4}\;m\;n\;\left(\frac{m^{2}}{L_{y}^{2}}+\frac{n^{2}}{L_{z}^{2}}\right)}\;\sin\;\left(\frac{m\pi y}{L_{y}}\right)\;\sin\;\left(\frac{n\pi z}{L_{z}}\right)$$

### 2.6. Computational Flow Dynamics Analysis

CFD simulations were conducted in ANSYS CFX to model flow through the perfusion chamber. The fluid domain was extracted from the chamber geometry, and a tetrahedral mesh was generated with refinement around the inlet, outlet, and orifice regions. The mesh consisted of a single 3D domain discretized into 104 Principal 2D regions to map its topology. The domain contained 307,091 nodes connected by 206,790 tetrahedral elements targeted to 1.27 mm quality with a minimum edge length of 0.39 mm. The fluid was modeled as incompressible and Newtonian, with properties approximating standard cell culture media at 37 °C. Density and viscosity values were based on calculated measurements or available literature. A steady-state, laminar flow regime was assumed. The inlet boundary condition was set as a mass flow rate of $4.0\times 10^{-7}$ kg/s, reflecting typical operating conditions for a driving pressure of 1 psi. Solutions were considered converged when residuals dropped below $1\times 10^{-6}$, and flow characteristics were evaluated through velocity contours, vector fields, and viscosity iso-surfaces to confirm full perfusion without separation.

### 2.7. Percent Degradation and Inter-Spheroid Distance Measurements

To calculate the percentage of hydrogel area degraded, bioengineered tissue patches were imaged on the Invitrogen EVOS microscope. Images were processed and analyzed in Fiji [9]. Areas corresponding to holes within the hydrogel matrix were measured, and the sum of these areas was divided by the total hydrogel matrix area to determine the percentage that had degraded. This analysis was performed on bioengineered tissue patches that were either perfused for 24 h or maintained under static conditions as a control. To determine the inter-spheroid distance, bioengineered tissue patches were similarly imaged and the distance of each spheroid to its closest neighboring spheroid was measured. Measurement analysis was conducted in Fiji by determining the length from the center of one spheroid to the center of the other. This was performed for every spheroid in the tissue patches.

### 2.8. Graphical and Statistical Analysis

R and Jupyter Notebook (Python) were used to create graphical representations. A two-tailed Student’s *t*-test was performed for statistical comparison. Flow cytometry results were analyzed and visualized in FlowJo™.

## 3. Results

**Figure 2:**
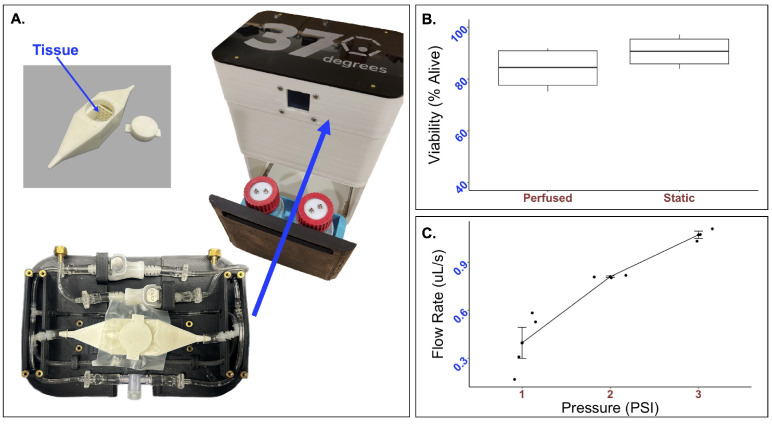
Real-time performance of the integrated perfusion system. (**A**) The integrated perfusion system (right) and custom try and bioreactor (left) takes up minimal space. To determine the compatibility of the system with live cells (**B**), 33k mMSC and mEC spheroids were formed and suspended in a mixture of thrombin and fibrin to form a tissue patch. Tissue patches were perfused for 24 h, and Live/Dead was analyzed through flow cytometry. No statistically significant difference in cell viability was observed. The average percent of live cells after 24 h was 83.99±8.47% while that of the static control was 90.596±6.39% (*p*-value = 0.26). To test the mechanical parameters of the FluidON, (**C**) volumetric flow rate was determined across a range of pressures.

The compatibility of the integrated FluidON system (Figure 2A) with live bioengineered tissue patch samples were determined. Bioengineered hydrogel tissue patches were created by embedding mMSC and mEC spheroids in fibrin and thrombin. The mixture then underwent gelation, forming a semi-solid hydrogel with spheroids embedded inside. The bioengineered tissue patch was then subject to perfusion in the FluidON.

Tissue viability was measured using flow cytometry. Bioengineered tissues were created using a vinyl specimen mold and subject to perfusion at 2 psi. The viability of samples perfused for 24 h was found to be 83.99±8.47% alive (n = 4). In comparison, the static samples were 90.59±6.39% alive (n = 4) (Figure 2B). A *t*-test returned a *p*-value of 0.2628(t=−1.2452,df=5.5816). As no statistically significant differences in cell viability were observed, the FluidON system is capable of low velocity perfusion without negatively impacting cell health.

To determine the FluidON’s system parameters, we measured the flow rate (*V*) across a range of pressures (Figure 2C). The volumetric flow rates, average flow velocities ($u_{avg}$), maximum flow velocities ($u_{max}$), maximum shear stress ($\tau_{max}$), and Reynolds number (*Re*) for each psi are given in Table 1. Calculations are described in detail in the methods section.

We found that each *Re* was much less than 2000, demonstrating that the flow is considered laminar at 1–3 psi. At 1 psi—the lower limit of the system which is most comparable to physiological interstitial flow—the *Re* was equal to 0.8. Thus, the system meets the condition (*Re* <1) where the fluid exhibits creeping flow—a property where viscous forces dominate the flow. This property simplifies the Navier-Stokes equation which can be used to obtain the 3D flow velocity profile.

**Figure 3:**
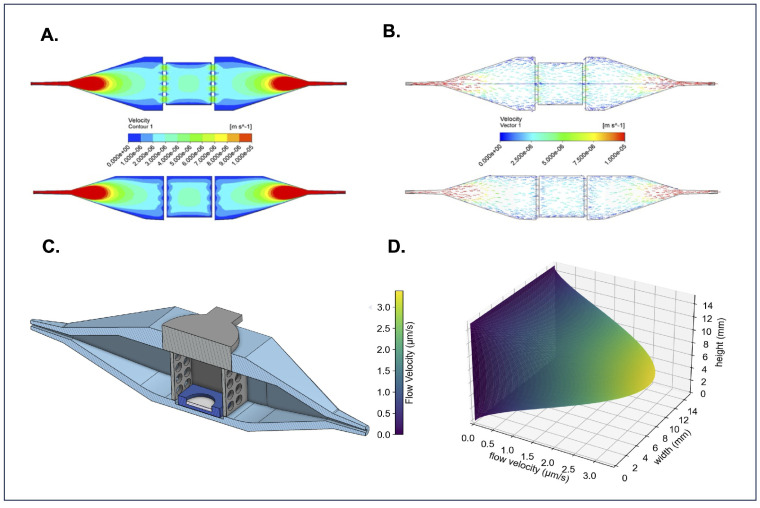
Overview of bioreactor computational fluid dynamics. (**A**) Top-down and side cross-sections of velocity contours demonstrate how the fluid accelerates and the range of flow velocities in every region of the bioreactor’s interior. (**B**) Top-down and side cross-sections of flow velocity vectors show that the flow is fully developed and parallel to the bioreactors walls in the tissue loading region. These CFD simulations can be compared to (**C**) a CAD cross-sectional model of the bioreactor for reference. (**D**) 3D flow velocity profile through the bioreactor’s sample-containing region.

Computational fluid dynamics (CFD) with an inlet volumetric flow rate of 0.4 μL/s was used to simulate the 3D flow velocity profile within the bioreactor. The flow velocity profiles show a roughly parabolic flow distribution with low-flow velocity boundary layers on the walls parallel to the *x*-axis (Figure 3A). The flow velocity in the center of the bioreactor appears to be fully developed with a $v_{max}$ between 3–4 μm/s, encompassing the quantity we calculated (3 < 3.38 < 4 μm/s) as the maximum flow velocity in Table 1. Likewise, velocity vectors confirm uniform flow distribution and laminar flow within the bioreactor’s center chamber, where tissues are housed (Figure 3B). A CAD model (Figure 3C) of the bioreactor is included to provide a structural reference for comparison with the CFD simulations. The Navier-Stokes equation under creeping flow conditions was used to obtain an approximation of the flow velocity profile, visualizing the speed fluid flows at every point in the tissue-containing center chamber (Figure 3D). The FluidON, therefore, can be categorized as a perfusion system targeting interstitial flow rates. Balanced fluid distribution, velocity, and viscosity within the tissue chamber ensure uniform perfusion of samples, supporting consistent nutrient delivery and helping promote tissue maturation.

Although hydrogels formed from fibrin and thrombin are highly biocompatible, they exhibit poor mechanical properties and are susceptible to rapid degradation [10]. Studies suggest that hydrogel degradation may be driven by cell-secreted matrix metalloproteinases, leading to the formation of holes or tears within the matrix [11]. Such structural deformities may compromise the integrity of bioengineered tissue patches, as spheroids can detach and fall out of the hydrogel before vascularization between spheroids can occur. Quantification of hydrogel degradation rates [12] can help assess material effectiveness.

**Figure 4:**
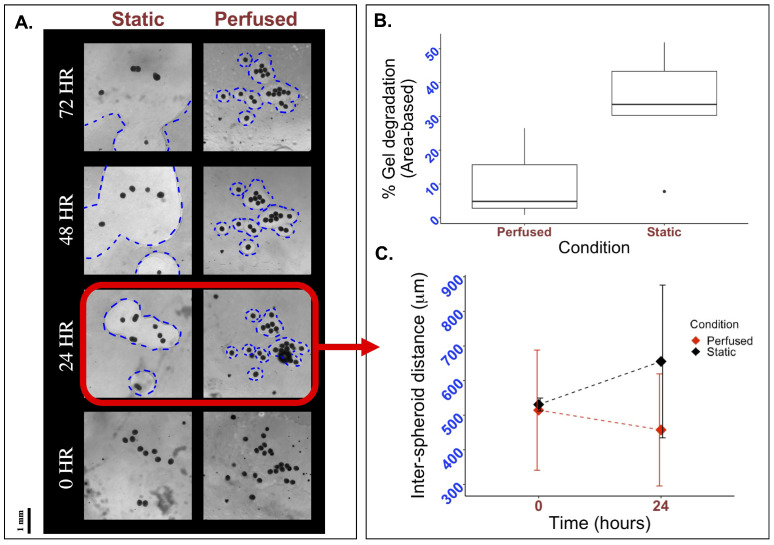
Effects of perfusion on gel degradation and spacial organization. To visually inspect the effects of perfusion, 33k mMSC and mEC spheroids were formed and suspended in a fibrin and thrombin mixture. This mixture was plated on a mold, perfused, and observed under a light microscope. (**A**) Holes form in the matrix as hydrogels degraded, with the perfused sample exhibiting smaller holes than the static control at all time points. Holes are outlined in blue for clarity. (**B**) Quantifying the area of the holes in Fiji, it is observed that the percentage of hydrogel degradation is decreased in perfused conditions. (**C**) The length from each spheroid to its closest neighbor was measured for all spheroids in the tissue sample at 0 and 24 h. Though inter-spheroid distance is initially similar, after 24 h the average distance between spheroids grows to 655.32 µm in static conditions while it decreases to 457.41 µm in perfused conditions.

To evaluate if perfusion using the FluidON system impacted hydrogel stability, we fabricated bioengineered tissue patches on glass and PDMS molds and subjected them to static or perfused conditions. Tissues were imaged using a light microscope, and hydrogel degradation was quantified through the area of holes formed within the matrix, as described in the Methods section. Visually, a greater degree of hydrogel degradation was observed under static conditions, with hole sizes (outlined in blue) increasing rapidly from 24 to 72 h relative to perfused samples (Figure 4A). Quantitative analysis of the percent area degraded after 24 h revealed that patches under static conditions (n = 5) on average degraded by 33.39±16.64%, while perfused patches (n = 3) degraded by 10.74±13.83%(t=0.091) (Figure 4B). As such, hydrogel degradation under perfused conditions was reduced compared to the static control.

Studies report that the distance between spheroids when embedded in hydrogels can have large impacts on spheroid behavior and subsequent cell vascularization [13]. To determine if perfusion with the FluidON caused any spatial changes in inter-spheroid distance, bioengineered tissue samples were perfused, and the distance between spheroids was measured. Inter-spheroid distances under perfused (n = 3) and static (n = 3) conditions were similar at 0 h, measured at 514.52±173.5μm and 530.85±18.78μm, respectively. After 24 h, the inter-spheroid distance decreased to 457.41 μm under perfused conditions, whereas it increased to 655.32 µm under static conditions (Figure 4C). Previous studies have reported that an intercellular distance of approximately 250 μm is optimal for promoting paracrine signaling, thereby enhancing cell growth and angiogenesis [10,14]. While optimal distances may vary depending on specific cell–cell interactions and cell types [15], perfusion reduced the inter-spheroid distance, bringing spheroids closer to the reported optimal spacing, which may promote enhanced vascularization. Taken together, perfusion demonstrates promising potential for enhancing bioengineered tissue fabrication.

**Figure 5:**
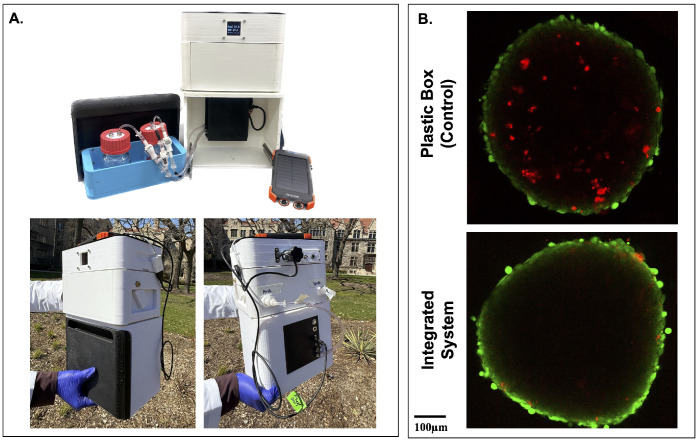
Transportation of tissues in the integrated system. (**A**) The FluidON and CultureON systems were powered by an external battery, enabling continuous operation during outdoor transportation. The system is shown open to highlight internal components (top), and the system’s front and back (bottom) were captured during transportation. (**B**) Following a 20-min walk, simulating real-world tissue transfer conditions, spheroids embedded within the tissue patch were stained using a Live/Dead assay, with green fluorescence indicating viable cells and red fluorescence indicating non-viable cells. Tissue patches transported in the integrated system exhibited more viable cells than the control.

To evaluate the feasibility of tissue transport, the integrated FluidON and CultureON were powered by an external 10,000 mAh battery (Blavor). This allowed for disconnection from all wall-mounted power sources and enabled transportation. The system successfully maintained both incubation and perfusion functions outdoors and under real-world conditions (Figure 5A). To test if transportation in the integrated system had positive benefits for tissue viability, engineered tissue patches were maintained within the device during a 20-min outdoor walk, simulating potential transfer between clinical or research facilities. A control was transported in a plastic box. No perfusion was performed to enable better comparison. Tissues were stained with LIVE/DEAD™ Viability/Cytotoxicity Kit before being imaged on the STELLARIS Confocal Microscope (Leica; Wetszler, Germany). Spheroids within tissue patches transported using the integrated system exhibited notably fewer dead cells, as indicated by reduced red ethidium homodimer-1 staining, compared to those transported in a standard plastic container (Figure 5B). These preliminary results suggest promising potential for the usage of the integrated system in supporting tissue stability during transportation.

## 4. Discussion

Perfusion serves as a promising tool to advance bioengineered tissue creation, and many unique systems have been introduced in recent years. One such is the organ-on-a-chip (OoC) model, which allows for biomimetic flow and the integration of key mechanical and electrical stimuli. However, because the system is constituted by microfluidic devices, it is tailored toward single organoids and microtissues rather than tissue constructs [16]. On the other hand, large organ perfusion systems house larger tissues or organs, but their greater flow velocity may harm tissue maturation [17]. Perfusion systems that target interstitial flow may combine features of both OoC and large organ perfusion; they often provide slow, biomimetic flow while offering space to support tissue constructs. Therefore, while OoC and large organ perfusion have their unique uses, interstitial flow provides an ideal niche for tissue growth and maturation.

The FluidON, introduced in this paper, targets medium interstitial flow rates to support 3D tissue formation. A study by Radisic et al. similarly details a perfusion system with interstitial flow. They were able to perfuse a tissue scaffold for 7 days at 0.5 mL/min, and they noted significant increases in viability following perfusion relative to their control [18]. A slower flow rate of 0.045 mL/min was used in our study, but the FluidON can be tailored to the desired flow rate by changing the air pump’s pressure. Likewise, though our experiment was only conducted for 24–72 h, the desired test length can be increased as long as there is sufficient media. Radisic et al. noted the positive effects of interstitial perfusion on cell viability. Though the FluidOn demonstrated no significant changes in cell viability after 24 h, with longer study times, we anticipate similar improvements in viability, reflecting the system’s potential to support cell survival and tissue growth.

Regardless of the price point or flow targeted, most established perfusion systems use an external pump system [19,20,21]. Additionally, long-term perfusion experiments also require co-usage with an incubator, further increasing the size burden of the perfusion system. Taken collectively, this limits the portability of perfusion systems, anchoring them to a laboratory bench and reducing broader applications.

There lacks a system able to portability maintain perfusion under incubating conditions for long durations. Our perfusion system may provide a potential remedy as it integrates perfusion and culturing in a construct weighing <10 lbs. In their study, Wolf et al. introduce a portable perfusion system—the VascuTrainer—which can achieve pulsatile flow ranging from 10 to 2000 mL/min with a battery life of approximately 25 h [22]. Though demonstrating positive results, the VascuTrainer operates at a much higher flow rate than the optimal velocity for interstitial flow. Additionally, it lacks the ability to support tissue culture conditions during transport, making it better suited to receiving organ implants than for tissue maturation. With the ability to perfuse and culture simultaneously in a compact and mobile system, the FluidON may target portable tissue maintenance better than other available counterparts.

While preliminary studies demonstrate FluidON’s promising application, as an initial prototype, limitations may arise. To begin, though our results show meaningful differences, they are of a short duration. Longer studies could demonstrate more robust results. For example, although significant branching between spheroids was not observed at the 24 h time point under either perfused or static conditions, extended experimentation may enable a better understanding of the influence of perfusion on cell growth and spheroid vascularization. Extended experimentation will also enable a better understanding of the influence of perfusion on cell growth and spheroid vascularization.

In addition, as the current FluidON version did not have built-in refrigeration, media bottles had to be maintained in an external container for experiments with long durations. While the media is warmed to 37 °C as it passes through the CultureON incubation environment before entering the bioreactor, both the fresh and waste media bottles were stored in an ice-filled container to maintain media quality. Other perfusion systems place the fresh media source in an incubator alongside the sample. However, prolonged exposure to warm conditions can degrade metabolites in the culture media, reducing its effectiveness. Integration of refrigeration into the FluidON reagent bay will allow for an easier experimental setup and allow for increased perfusion durations.

Additionally, while the system pressure and drive voltage can be easily examined through an external monitor, new additions can be made to further improve the capabilities of the system. Precision flow sensors situated at crucial junctions in the system could enable real-time monitoring of the internal fluidic performance of the perfusion circuit. Moreover, the development of a live video microscopy system is underway, which could potentially reveal new insights into bioengineered tissues. Although most perfusion studies run continuously over time, analysis is typically limited to discrete time points, rather than capturing real-time data. Integrating a live video microscopy system into the CultureON and FluidON would help us understand cell migration patterns in real-time to better understand how perfusion impacts bioengineered tissues.

## 5. Conclusions

In this study, we introduced a novel and integrated portable perfusion system. Composed of the CultureON, the FluidON, a custom-designed bioreactor tray, and a bioreactor, the system is capable of maintaining continuous fluid flow at 37 °C and 5% CO_2_. While this environment is similar to that of standard incubators, a significant difference is that the total weight of the integrated perfusion system is <10 lbs and requires no external gas lines or wall-mounted power sources, facilitating easy transportation of tissue samples. Using a high-impedance fluidic resistor, the system achieves low flow velocities similar to interstitial flow. A 1 cm^3^ tissue scaffold can be perfused, expanding its utility from previously reported OoC and single-spheroid perfusion systems, which are typically limited to smaller-scale constructs. Our findings highlight the perfusion system’s potential to enhance tissue engineering outcomes. Cell viability remained unaffected relative to the control, suggesting that the system supports cell health and is biocompatible. Additionally, enhanced spatial organization of spheroids and reduced hydrogel matrix degradation supported the development of improved tissue patches. Currently, the FluidON system is focused on bioengineered tissue perfusion, however, additional applications can be envisioned. Given its portable size and capacity to sustain incubation and perfusion, the integrated system could potentially be extended toward broader sample transportation and more. While further studies must be conducted, this novel perfusion system demonstrates considerable promise for tissue engineering and related applications.

## Figures and Tables

**Table 1:** Perfusion Parameters of the FluidON.

Drive Pressure (psi)	*V* (μL/s)	$u_{\mathrm{avg}}$ (μm/s)	$u_{\max}$ (μm/s)	*Re*
1	0.40±0.19	1.69±0.83	3.38±1.66	0.80
2	0.81±0.0083	3.5±0.035	7±0.07	3.30
3	1.1±0.040	4.6±0.17	9.2±0.34	4.34

## Data Availability

The raw data supporting the conclusions of this article will be made available by the authors on request. Further inquiries can be directed to the corresponding author.
